# Effect of the plant-based hemostatic agent Ankaferd Blood Stopper® on the biocompatibility of mineral trioxide aggregate

**DOI:** 10.1186/s12903-016-0302-0

**Published:** 2016-10-11

**Authors:** Muzaffer Emir Dinçol, Hakan Ozbas, Bulent Yılmaz, Handan Ersev, Selcuk Gokyay, Vakur Olgac

**Affiliations:** 1 Faculty of Dentistry, Department of Endodontics, Istanbul University, 34093 Fatih, Istanbul, Turkey; 2 Institute of Oncology, Department of Tumor Pathology and Cytology, Istanbul University, 34093 Fatih, Istanbul, Turkey

**Keywords:** Ankaferd blood stopper, Biocompatibility, Mineral trioxide aggregate

## Abstract

**Background:**

Due to the detrimental effect of blood contamination on the physico-chemical properties of mineral trioxide aggregate (MTA), obtaining an effective hemostasis in the surgical crypt during apical surgery is of paramount importance. The purpose of this in vivo study was to analyze the effect of Ankaferd Blood Stopper® (ABS) contamination on the biocompatibility of MTA.

**Methods:**

Forty of 56 Wistar–Albino rats were divided randomly and equally into two groups (MTA and MTA-ABS) according to whether or not a hemostatic agent was used. The remaining 16 rats were designated as the control group. Rats in the experimental groups received freshly mixed MTA-Angelus in polyethylene tubes, which were inserted into monocortical bore holes created in their tibias. In the MTA-ABS group only, 0.5 mL of ABS solution was administered topically on the defect sites followed by implantation of MTA tubes. Inflammation, foreign-body reaction (FBR), necrosis, fibrosis, and new bone formation (NBF) were studied 7, 30, 60, and 90 days after implantation.

**Results:**

On day7, statistically significant differences were found in tissue reactions with regard to NBF and necrosis (*p* = 0.044 and *p* = 0.024, respectively), the latter being observed in 40 % of the samples only in the MTA-ABS group. Slight inflammation in all groups was confined to day-7 only. Mild necrosis was present in the MTA-ABS group only on day-7. Severity of the foreign body reaction and fibrosis was limited. New bone formation increased gradually over time in all groups, reaching a maximum on day-90.

**Conclusions:**

MTA and ABS-contaminated MTA are equally biocompatible. ABS does not impair the properties of MTA.

**Electronic supplementary material:**

The online version of this article (doi:10.1186/s12903-016-0302-0) contains supplementary material, which is available to authorized users.

## Background

Periapical surgery is commonly comprised of root-end resection, apical cavitation, and root-end filling to seal the communication between the periapical tissues and the root canal system [1]. An “ideal” root-end filling material should have properties like; easy handling, good biocompatibility and apical sealability at the root apex to prevent the egress of bacteria, and bacterial byproducts [2, 3]. It should also be insoluble in body fluids such as blood, saliva, and tissue fluid [4, 5]. Unfortunately, there is no ideal material that fulfills all of these requirements.

Various root-end filling materials have been suggested [6]. The calcium silicate-based compound mineral trioxide aggregate (MTA) has been compared with other root-end filling materials and hypothesized to be a “near-to-ideal substance” [7]. MTA is used widely in root-end filling, pulp capping, perforation repair, and in the treatment of immature apices. MTA is employed in these conditions because of its biocompatibility, antibacterial nature, ability to set in moist environments, and because it acts as a potential seal against bacterial penetration [8].

In endodontic surgery, blood comes into contact with (and often becomes incorporated into) MTA during or after its placement. This contamination has detrimental effects on its surface microhardness, compressive strength, bond strength and marginal adaptation [9]. Blood contamination of unset MTA adversely affects the setting reaction and mechanical strength of the material [10]. When considering the clinical uses of MTA, its physical properties are important factors in achieving a good seal between the root canal and the external surface of the root [9]. Therefore, in using MTA, attempts should be made to control bleeding [9, 10].

Various local hemostatic agents have been used in oral and maxillofacial surgery [11]. Ankaferd Blood Stopper® (ABS) (Ankaferd Sağlık ürünleri A.Ş., Istanbul, Turkey) is an extract from medicinal plants that have been used for centuries in Turkish traditional medicine as a hemostatic agent. ABS consists of a standardized mixture of several plants: *Urtica dioica* (6.0 g/100 ml)*, Vitis vinifera* (8.0 mg/100 ml)*, Alpinia officinarum* (7.0 mg/100 ml)*, Thymus vulgaris* (5.0 mg/100 ml) and *Glycyrrhiza glabra* (7.0 mg/100 ml) [12]. Each of these plants has an impact on cellular proliferation, vascular dynamics, angiogenesis, blood cells, cell mediators and endothelia [13]. Each constituent, if used separately, has interesting biologic effects. *U.diocia* can elicit vasorelaxation (mediated by the release of endothelial nitric oxide and the opening of potassium channels) as well as a negative inotropic action. *Vitis vinifera* exerts anticarcinogenic and antiatherosclerotic effects. *Alpina officinarum* inhibits nitric oxide production in macrophages. *Thymus vulgaris* has antioxidative actions such as prevention of peroxidation. *Glycyrrhiza glabra* has anti-inflammatory, antithrombin, antiplatelet, antioxidant, antiatherosclerotic, and anticarcinogenic effects [13, 14]. In addition, ABS has been found to be as effective as Surgicel® for achieving hemostasis in animal models [15, 16]. Moreover, ABS can be used to create a blood-free tooth surface during application of brackets on surgically exposed impacted teeth [13, 14]. ABS has also been used successfully for: dental extractions in patients undergoing antithrombotic treatment with acetylsalicylic acid and warfarin sodium without interrupting and/or altering the anticoagulant regimens [17]; in patients with hemorragic diathesis [18]; and in the treatment of acute and profuse bleeding of the gastrointestinal tract [19].

Reactions in tissues caused by hemostatic agents are important for the reliability of the materials used [20, 21]. Although ABS is widely used in endodontic surgery as a hemostatic agent [13], data regarding to its effects on root-end filling materials in animal experiments (devised for the investigation of their biocompatibility) are lacking. Thus, the aim of the present in vivo study was to evaluate and compare the effect of ABS on the early and long-term tissue reactions of MTA in a wound healing model at rat tibias. This is the first histopathologic study to ascertain whether ABS, when used as an hemostatic agent, affects the biocompatibility of MTA.

## Methods

### Animal care and sample grouping

The study was carried out in various departments within Istanbul University (Istanbul, Turkey). Care and use of laboratory animals was according to the criteria set by the Committee on Animal Research within Istanbul University (protocol number: 43/09). Rats were obtained from the Center of Experimental Medical Research of Istanbul University.

Fifty-six Wistar–Albino male rats (4–6 months, 250–300 g) were housed in groups of four per cage, fed standard pellets and water in a temperature-regulated room (22 °C, 55 % humidity, 12-h light–dark cycle), and allowed to mobilize freely. Forty rats were divided randomly and equally into two experimental groups according to whether or not a hemostatic agent was used. The remaining 16 rats were designated as the control group.

### Intraosseous surgical procedure

Each animal underwent a general anesthetic procedure by the injection of ketamine hydrochloride (10 %; Alfamine®; 15 mg/kg body weight, i.m.) and xylazine hydrochloride (2 %; Alfazyne®; 60 mg/kg, i.m.). After shaving the left and right hind limbs, rats were positioned in the ventral decubitus position. Antisepsis (10 % povidone-iodine solution (Isosol®, Central Laboratory, Istanbul)) was carried out over shaved areas. An incision (≈15 mm) was made at the lateral aspect of the anterior border of the tibia until the tibia was reached. After reflection of the periosteum, a monocortical circular defect (diameter, 1.5 mm) was created at the anterior portion of the diaphysis of both tibias until the bone marrow was reached. These defects were drilled using a round carbide burr in a low-speed handpiece under constant irrigation with physiologic (0.9 %) sterile saline solution to prevent overheating and necrosis of bone tissue.

MTA-Angelus (Angelus Indústria de Produtos Odontológicos, Londrina, Brazil) was mixed according to the manufacturer’s instructions. Then, the freshly mixed cement was introduced into sterile polyethylene (non-toxic, intravenous catheter, 18 G) tubes (external diameter, 1.2 mm; length, 10 mm; sealed at one end) with the aid of a syringe having a needle compatible with the inner diameter of the tubes. Empty tubes were used for rats in the control group. Tubes were introduced so that they projected into the bone-marrow compartment and their lateral walls were parallel to the long axis of the tibia with the closed ends pointing towards the knee joint [22].

In the MTA-ABS group, 0.5 mL of ABS solution was administered topically on the defect sites using an insulin syringe, after which the tubes containing MTA-Angelus were implanted into the right tibias. In the MTA group, local hemostasis was achieved with direct packing with gauze on the defect sites, and then the tubes containing MTA-Angelus were implanted into the left tibias. In the control group, local hemostasis was maintained in the same manner as that in the MTA group, and then empty tubes were inserted into both tibias. The muscle layer was sutured with absorbable 4/0 gut sutures, and the skin closed with 3/0 silk thread (M.E.D). No antibiotic was given and the rats were observed postoperatively. All animals survived the indicated time periods without complications.

### Histological procedures

After each time period (7, 30, 60, and 90 days), 14 rats were sacrified with an overdose of ketamine hydrochloride (50 mg/kg). Entire tibias were isolated by gross dissection, fixed in 10 % formalin for 24 h, decalcified in 20 % formic acid, and processed for embedding in paraffin. Sections were cut parallel to the long axis of the tubes at 7 μm, mounted on slides, and stained with hematoxylin and eosin (H&E). Slides were analyzed and photographed at different magnifications in five areas with a light microscope (Eclipse E600; Nikon, Tokyo, Japan) coupled with a digital camera, by an oral pathologist (V.O) blinded to the study protocol.

### Morphometric analysis

To estimate the level of inflammation and recovery in bone-marrow tissue adjacent to the open end of each tube, all sections were analyzed to evaluate inflammation, FBR, fibrosis, NBF, and necrosis. When evaluating FBR, the presence of foreign body giant cells and when evaluating inflammation, that of lymphocytes and plasmocytes were considered as basis. In the NBF, the trabecular, web-like woven bone or the thin lamellar bone tissue which tends to cover the area adjacent to the open end of the tube were considered as essentials. The bone trabecules without osteoblasts around, were considered as structures belonging to pristine bone and were neglected. While evaluating necrosis, at the open end of the tubes, the loss of the characteristic staining capability of cellular nuclei and disappearance of the details of cellular morphology, forming the classical appearance of cell death was considered as necrosis. Scores for these reactions (except for necrosis) were estimated by measuring the magnitude of the areas occupied by the tissue reactions in a standardized microscopic field at x40 magnification at the open end of the tubes by using an image analysis software (Olympus analySİS FIVE, Tokyo, Japan). A cell percentage of 0–25 % was scored semiquantitatively as “none” (0), 25–50 % as “mild” (1), 50–75 % as “moderate” (2), and 75–100 % as “severe” (3), [6, 13]. Scores for necrosis were determined according to its presence (1) or absence (0), [6].

### Statistical analysis

Values for the severity of inflammation, FBR, fibrosis as well as the level of NBF were compared using the Kruskal–Wallis and the Mann–Whitney U non-parametric tests. Values for the presence or absence of necrosis were compared with the Fisher–Freeman–Halton test. Analyses were done using SPSS v22.0 (IBM, Armonk, NY, USA) with (*p* < 0.05) being considered significant.

## Results

**Fig. 1:**
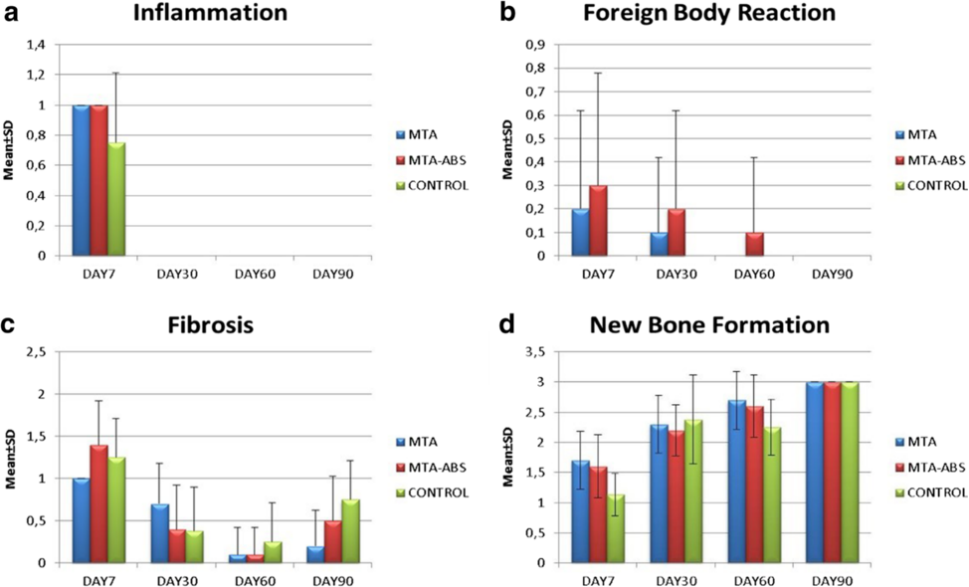
The mean and standart deviation of reaction scores in all groups at time intervals. **a** Inflammation; **b** Foreign body reaction; **c** Fibrosis and **d** New bone formation

**Fig. 2:**
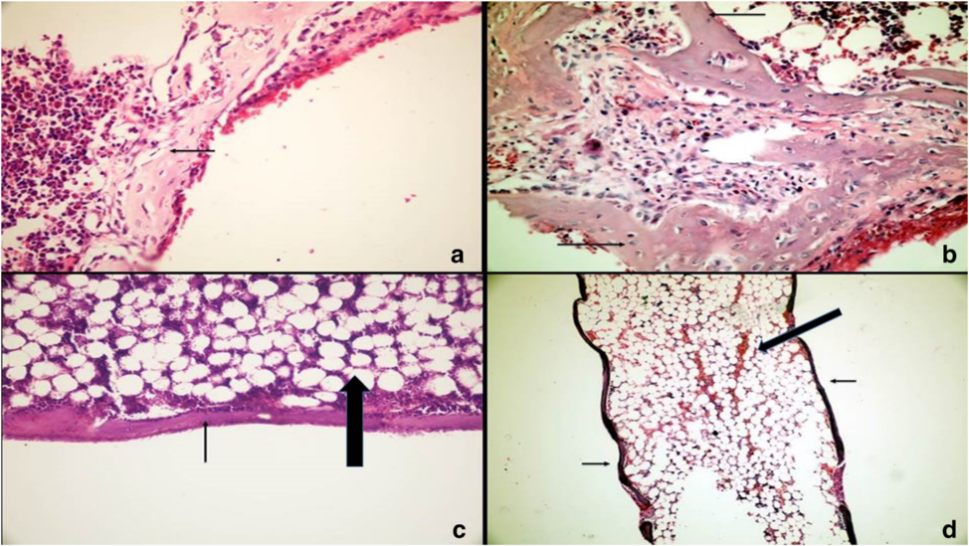
Tissue reactions in the control group. 7 days: **a** Thin trabeculae of new bone (*arrow*) was observed at the open end of the tube (H&E,x400). 30 days: **b** New bone formation (*arrows*) adjacent to the tube end (H&E,x400). 60 days: **c** New bone became lamellar (*arrow*) at the open end of the tube. Normocellular bone marrow (*bold arrow*) is seen under the bone (H&E,x200). 90 days: **d** Lamellar bone (*arrows*) surrounding fatty bone marrow (*bold arrow*) getting into the tube (H&E,x40)

**Fig. 3:**
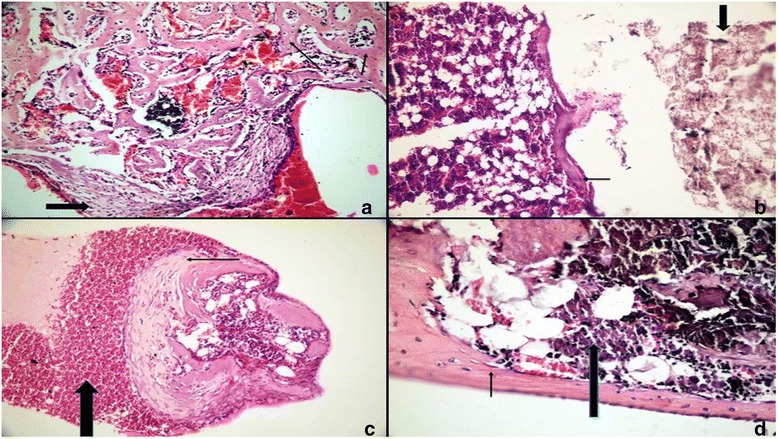
Tissue reactions in the MTA group. 7 days: **a** Fibrous connective tissue (*bold arrow*) and new bone trabeculae (*arrows*) were seen at the open end of the tube (H&E,x200). 30 days: **b** Lamellar bone formation (*arrow*) and residual MTA (*bold arrow*) were observed (H&E,x100). 60 days: **c** New bone formation (*arrow*) and normocellular bone marrow (*bold arrow*) were seen at the open end (H&E,x200). 90 days: **d** Mature lamellar bone (*arrow*) and normocellular bone marrow (*bold arrow*) (H&E,x200)

**Fig. 4:**
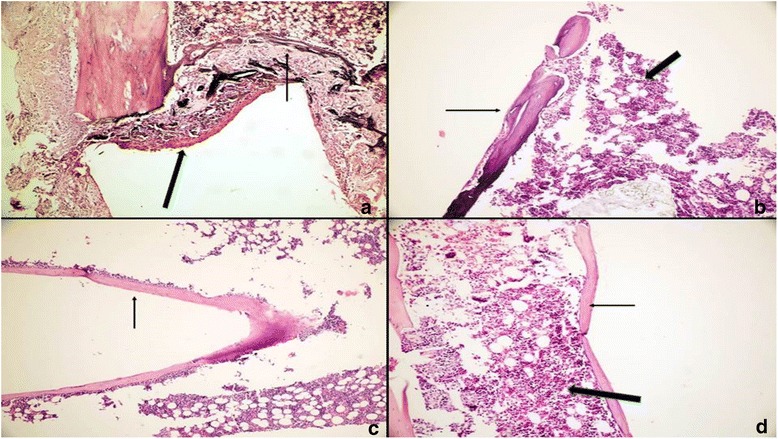
Tissue reactions in the MTA-ABS group. 7 days: **a** Fibrous band of connective tissue (*bold arrow*) and tiny bone trabeculae (*arrow*) adjacent to the open end (H&E,x100). 30 days: **b** Lamellar bone (*arrow*) and bone marrow (*bold arrow*) at the open end of the tube (H&E,x200). 60 days: **c** Mature lamellar bone (*arrow*) surrounding the open end of the tube (H&E,x200). 90 days: **d** Mature lamellar bone (*arrow*) and bone marrow (*bold arrow*) (H&E,x100)

At the end of all time periods, wound healing was satisfactory for all rats and all implanted biomaterials remained in situ. Scores and percentages for inflammation, FBR, necrosis, fibrosis, and NBF in all groups are shown in Tables 1–2 and illustrated in Fig. 1 (see Additional file 1, for a detailed version of scores as raw data). Representative histologic images of specimens in all study groups at all times are illustrated in Figs. 2, 3 and 4.

**Table 1:** Comparison of results belonging to the study groups at study intervals for the 5 events assessed

Histologic events	Experimental periods	Study groups			*p*
		MTA	MTA-ABS	Control	
Inflammatory response (Mean ± SD)	7 days	1 ± 0	1 ± 0	0,75 ± 0,46	**0,075**
	30 days	0 ± 0	0 ± 0	0 ± 0	**1,000**
	60 days	0 ± 0	0 ± 0	0 ± 0	**1,000**
	90 days	0 ± 0	0 ± 0	0 ± 0	**1,000**
	*p*	**0,001** ^*^	**0,001** ^*^	**0,001** ^*^	
Foreign body reaction (Mean ± SD)	7 days	0,2 ± 0,42	0,3 ± 0,48	0 ± 0	**0,262**
	30 days	0,1 ± 0,32	0,2 ± 0,42	0 ± 0	**0,407**
	60 days	0 ± 0	0,1 ± 0,32	0 ± 0	**0,407**
	90 days	0 ± 0	0 ± 0	0 ± 0	**1,000**
	*p*	**0,276**	**0,281**	**1,000**	
Fibrosis (Mean ± SD)	7 days	1 ± 0	1,4 ± 0,52	1,25 ± 0,46	**0,097**
	30 days	0,7 ± 0,48	0,4 ± 0,52	0,38 ± 0,52	**0,300**
	60 days	0,1 ± 0,32	0,1 ± 0,32	0,25 ± 0,46	**0,603**
	90 days	0,2 ± 0,42	0,5 ± 0,53	0,75 ± 0,46	**0,071**
	*p*	**0,001** ^*^	**0,001** ^*^	**0,004** ^*^	
New bone formation (Mean ± SD)	7 days	1,7 ± 0,48	1,6 ± 0,52	1,13 ± 0,35	**0,044** ^***^
	30 days	2,3 ± 0,48	2,2 ± 0,42	2,38 ± 0,74	**0,681**
	60 days	2,7 ± 0,48	2,6 ± 0,52	2,25 ± 0,46	**0,154**
	90 days	3 ± 0	3 ± 0	3 ± 0	**1,000**
	*p*	**0,001** ^*^	**0,001** ^*^	**0,001** ^*^	
Necrosis [n(%)]	7 days	0(%0)	4(%40)	0(%0)	**0,024** ^***^
	30 days	0(%0)	0(%0)	0(%0)	**-**
	60 days	0(%0)	0(%0)	0(%0)	**-**
	90 days	0(%0)	0(%0)	0(%0)	**-**
	*p*	-	**0,009** ^**^	-	

*MTA* Mineral trioxide aggregate, *ABS* Ankaferd blood stopper, *SD* Standart deviation; ^*^Significant difference within the groups at *p* < 0.05; ^**^Significant difference within the groups at *p* < 0.01; ^***^Significant difference among the groups at *p* < 0.05; Bold data confines only to *p* values both within or among the groups

**Table 2:** Distribution of new bone formation and fibrosis grades in the study groups at study intervals

Histologic events	Study groups	Intensity of reaction	Experimental periods			
			7 days	30 days	60 days	90 days
New Bone Formation	MTA (%)	Severe	0	30	70	100
		Moderate	70	70	30	0
		Mild	30	0	0	0
		None	0	0	0	0
	MTA-ABS (%)	Severe	0	20	60	100
		Moderate	60	80	40	0
		Mild	40	0	0	0
		None	0	0	0	0
	Control (%)	Severe	0	50	25	100
		Moderate	12.5	37.5	75	0
		Mild	87.5	12.5	0	0
		None	0	0	0	0
Fibrosis	MTA (%)	Severe	0	0	0	0
		Moderate	0	0	0	0
		Mild	100	70	10	20
		None	0	0	0	0
	MTA-ABS (%)	Severe	0	0	0	0
		Moderate	40	0	10	0
		Mild	60	40	0	50
		None	0	0	0	0
	Control (%)	Severe	0	0	0	0
		Moderate	25	0	0	0
		Mild	75	37.5	25	75
		None	0	0	0	0

*MTA* Mineral trioxide aggregate, *ABS* Ankaferd blood stopper

An inflammatory reaction was observed in the MTA, MTA-ABS and the control groups only at the first study interval on day 7, being absent in the remaining study intervals. No such reaction was present in any of the groups at 30, 60, or 90 days. There were no significant differences (*p* > 0.05) among the study groups on day-7, whereas significant differences (*p* < 0.05) were detected when day-7 was compared with the inflammation at 30, 60, or 90 days.

At the open ends of the inserted tubes, macrophages in the connective tissue were detected to phagocyte the implanted biomaterial at the junctional area, where the connective tissue came in touch with the biomaterial, forming foreign body giant cells which induced a foreign body reaction and which was observed in two samples in the MTA group and in three samples in the MTA-ABS group on day 7; in one sample in the MTA and in two samples in the MTA-ABS groups on day 30; and only in one sample in the MTA-ABS group on day 60. Extent of FBRs among groups was not significant (*p* > 0.05) at 7, 30, 60, or 90 days. At these intervals, a FBR was not present in the control group. There were no significant differences (*p* > 0.05) among time periods in any group.

There was no necrosis in the MTA group or the control group on day-7. Necrosis was present in the MTA-ABS group only on day-7 and differences between this group and the other two groups were significant (*p* < 0.05). A significant difference (*p* < 0.01) was present only in the MTA-ABS group on day-7 among the time intervals used.

With regard to the severity of fibrosis, no significant differences (*p* > 0.05) were present among MTA, MTA-ABS, and control groups at 7, 30, 60, or 90 days. However, significant differences (*p* < 0.05) were detected among time periods in all groups. Distribution of percentages of fibrosis in each study group at time intervals is shown in Table 2.

Intensity of NBF showed a significant difference (*p* < 0.05) among the study groups only on day-7. In the control group, the prevalence of NBF was significantly lower than either of the MTA and the MTA-ABS groups, but differences in NBF between these two groups were not significant at 30, 60, or 90 days. Comparison of the prevalence of NBF among groups showed significant differences (*p* < 0.05) during time periods, but the prevalence of NBF of specimens in each group varied with time (Table 2).

## Discussion

Intraosseous models provide an appropriate environment for in vivo testing of the biomaterials used in endodontic surgery. The biomaterials to be tested for biocompatibility by intraosseous implantation are usually placed into the tibias and femurs of small laboratory animals like rats and guinea-pigs [13, 18, 22–24]. When a root end filling material is placed into the root end cavity, it comes into contact with periradicular tissues. Therefore, the reaction of bone to a biomaterial allows one to evaluate its biocompatibility. Thus, we have preferred to test the osseous reaction to our materials using the tibias of Wistar rats in a similar manner as reported in the endodontic literature [3, 7, 13, 22, 23]. Also in these studies, one can see that the study periods vary from author to author and there are no standart time points. If one desires immediate or early reactions, 7-day, 15-day and 30-day intervals are chosen. For long term reactions, the study intervals like 60 and 90-days can be added. Olsen et al. [22] have selected 7-14-21-56-100 days for time points.

A mild temporary inflammatory reaction was limited to 7 days in all groups. The inflammatory reaction in the control group was attributed only to the trauma produced during the tube placement [25]. However, in MTA and MTA-ABS groups, the high pH (owing to the release of calcium hydroxide when MTA was mixed with water), production of heat by an exothermic reaction during the setting of MTA, release of proinflammatory cytokines such as interleukin-1 and 6 during the process, and the aluminium content of the MTA, had additive effects on the inflammation caused by the surgical trauma [2, 3, 26].

Necrosis can be caused by almost any type of severe injury. In this study, tissue necrosis was only found, on day 7, in 4 samples of the MTA-ABS group where the possible cause may be the irritating effect of the material itself and the contamination with ABS. The disappearance of necrosis on day 30 may be related to the elimination of the acute irritating potential of the implanted material. Nevertheless, it has been shown that ABS is not toxic or allergenic, but has antibacterial and anti-inflammatory properties. ABS did not cause necrosis in bone tissue or defect areas according to histology. Moreover, ABS has been shown to reduce the prevalance of inflammation and necrosis in animal experiments [13, 18, 27]. Thus, ABS may not have caused this inflammation.

A FBR can occur if a foreign material is inserted into tissues. Fibroblasts proliferate and surround the foreign body. However, the latter is insoluble or indigestible by phagocytes, and is isolated by connective tissue [28]. FBRs were documented in the MTA and the MTA-ABS groups. In addition to all these, the FBR in the MTA-ABS group was found to be higher than that of the MTA group, the difference being statistically insignificant which may be attributed to the limited number of rats allowed due to ethical considerations. In the control group, a FBR was not detected at any time interval. In our opinion, the FBR in these two groups was owing to modified cells (epitheloid cells), remnants, constituents of mineral origin (waxy or oily remains), or complex polysaccharides and polymers [29].

Fundamentally, a connective-tissue reaction is a response of tissues to injury, and the damage tends to be repaired [29]. Severity of fibrosis among MTA, MTA-ABS, and control groups was very mild and was not significant at any time interval. Hence, the inflammation observed on day-7 was not persistent, and subsided in a short time. A stepwise increase in NBF was detected in all groups but a significant difference was not observed at any interval. In the control group, NBF increased but to a lesser extent. Distribution of NBF variations at time intervals is shown in Table 2.

All materials evoked an inflammatory reaction on day-7, this finding is in accordance with those of other studies [2, 3, 7]. Slight inflammation on day-7 and its absence at 30 days suggest that MTA and ABS-contaminated MTA had acceptable biocompatibility [8]. Absence of inflammation followed by NBF in surrounding areas owing to their osteoconductive properties, seems to be the best proof of biocompatibility for MTA and ABS-contaminated MTA [23].

An acceptable level of biocompatibility of a material ranges between no inflammatory response upon implantation, to an initial severe response that diminishes over time [30]. A biocompatibility scale has been devised by Moretton et al. [2] comprising of four levels, each associated with the severity of inflammation and its variation over time. This scale is used to assess the clinical relevance of findings of implantation experiments in animals whereby the first three levels denote an acceptable degree of biocompatibility. In the present study, the inflammatory response was mild and confined to the first week. According to the biocompatibility scale, MTA and ABS-contaminated MTA were biocompatible materials with almost identical effects on neighboring tissues. This mild response shows that both materials had better biocompatibility than those reported by Moretton et al. [2] and other scholars [3, 31–34]. The “gold standard” of biocompatibility is bone formation [24]. The absence of inflammation, together with a greater amount of hard-tissue formation against MTA and ABS-contaminated MTA, indicates their biocompatibility [8, 23].

MTA and ABS-contaminated MTA had similar effects throughout this 90-day study. Accordingly, ABS neither increased nor decreased the characteristic features of MTA but exerted a hemostatic effect. The “ideal” hemostatic agent is one that is safe, sterile, easy to apply, visible in the cavity, easy to remove, non-irritant, dissolves and degrades without interfering with periapical healing, and is an integral part of current surgical protocols [11, 17, 21].

Interestingly, ABS possesses features or functions not dissimilar to those mentioned above. Emes and colleagues, using an in vitro cell-culture model of rat fibroblasts, [35] stated that ABS, fibrin glue, and tranexamic acid may negatively affect tissue healing. However, other scholars have stated that ABS increases NBF in the early bone-healing period [13, 18]. Our findings show that ABS neither promoted nor inhibited the NBF of MTA. In our opinion, this potent hemostatic agent may be used in endodontic surgery owing to its biologic properties. However, further research is needed to clarify its beneficial effects and to detect potential unfavorable features.

## Conclusions

Consequences of contamination of MTA with a new plant-based hemostatic agent, ABS, was investigated in an in vivo system to assess if it had an impact on MTA function. ABS had no deleterious effects on the mode of action of this root-end filling material.

## Additional file

Additional file 1: The raw data. (DOCX 25 kb)

## Abbreviations

ABS: Ankaferd Blood Stopper
FBR: Foreign body reaction
MTA: Mineral trioxide aggregate
NBF: New bone formation
