# Supplementary material for: Effect of the plant-based hemostatic agent Ankaferd Blood Stopper® on the biocompatibility of mineral trioxide aggregate
Source: BMC Oral Health. 2016 Oct 11;16:111. doi: 10.1186/s12903-016-0302-0 (PMC5057429; doi:10.1186/s12903-016-0302-0)
Supplement: Additional file 1: — The raw data. (DOCX 25 kb) [file 12903_2016_302_MOESM1_ESM.docx]

| **Scores of tissue reactions for MTA group on day-7** | **INFLAMMATION** | **NECROSIS** | **FOREIGN BODY REACTION (FBR)** | **FIBROSIS** | **NEW BONE FORMATION (NBF)** |
| --- | --- | --- | --- | --- | --- |
| **Day 7- MTA_1_** | **1** | **0** | **0** | **1** | **2** |
| **Day 7- MTA_2_** | **1** | **0** | **0** | **1** | **2** |
| **Day 7- MTA_3_** | **1** | **0** | **0** | **1** | **2** |
| **Day 7- MTA_4_** | **1** | **0** | **1** | **1** | **1** |
| **Day 7- MTA_5_** | **1** | **0** | **0** | **1** | **2** |
| **Day 7- MTA_6_** | **1** | **0** | **0** | **1** | **1** |
| **Day 7- MTA_7_** | **1** | **0** | **0** | **1** | **2** |
| **Day 7- MTA_8_** | **1** | **0** | **0** | **1** | **2** |
| **Day 7- MTA_9_** | **1** | **0** | **0** | **1** | **2** |
| **Day 7- MTA_10_** | **1** | **0** | **1** | **1** | **1** |

| **Scores of tissue reactions for MTA-ABS group on day-7** | **INFLAMMATION** | **NECROSIS** | **FOREIGN BODY REACTION (FBR)** | **FIBROSIS** | **NEW BONE FORMATION (NBF)** |
| --- | --- | --- | --- | --- | --- |
| **Day 7- MTA-ABS_1_** | **1** | **0** | **1** | **2** | **2** |
| **Day 7- MTA-ABS_2_** | **1** | **0** | **0** | **1** | **2** |
| **Day 7- MTA-ABS_3_** | **1** | **1** | **0** | **2** | **1** |
| **Day 7- MTA-ABS_4_** | **1** | **1** | **0** | **1** | **1** |
| **Day 7- MTA-ABS_5_** | **1** | **0** | **0** | **1** | **2** |
| **Day 7- MTA-ABS_6_** | **1** | **0** | **1** | **1** | **2** |
| **Day 7- MTA-ABS_7_** | **1** | **0** | **1** | **2** | **2** |
| **Day 7- MTA-ABS_8_** | **1** | **0** | **0** | **1** | **2** |
| **Day 7- MTA-ABS_9_** | **1** | **1** | **0** | **2** | **1** |
| **Day 7- MTA-ABS_10_** | **1** | **1** | **0** | **1** | **1** |

| **Scores of tissue reactions for CONTROL group on day-7** | **INFLAMMATION** | **NECROSIS** | **FOREIGN BODY REACTION (FBR)** | **FIBROSIS** | **NEW BONE FORMATION (NBF)** |
| --- | --- | --- | --- | --- | --- |
| **Day 7- EMPTY TUBE_1_** | **0** | **0** | **0** | **1** | **1** |
| **Day 7- EMPTY TUBE_2_** | **1** | **0** | **0** | **2** | **1** |
| **Day 7- EMPTY TUBE_3_** | **1** | **0** | **0** | **1** | **1** |
| **Day 7- EMPTY TUBE_4_** | **1** | **0** | **0** | **1** | **2** |
| **Day 7- EMPTY TUBE_5_** | **1** | **0** | **0** | **1** | **1** |
| **Day 7- EMPTY TUBE_6_** | **1** | **0** | **0** | **1** | **1** |
| **Day 7- EMPTY TUBE_7_** | **0** | **0** | **0** | **1** | **1** |
| **Day 7- EMPTY TUBE_8_** | **1** | **0** | **0** | **2** | **1** |

| **Scores of tissue reactions for MTA group on day-30** | **INFLAMMATION** | **NECROSIS** | **FOREIGN BODY REACTION (FBR)** | **FIBROSIS** | **NEW BONE FORMATION (NBF)** |
| --- | --- | --- | --- | --- | --- |
| **Day 30- MTA_1_** | **0** | **0** | **0** | **1** | **2** |
| **Day 30- MTA_2_** | **0** | **0** | **0** | **1** | **2** |
| **Day 30- MTA_3_** | **0** | **0** | **0** | **1** | **2** |
| **Day 30- MTA_4_** | **0** | **0** | **0** | **0** | **3** |
| **Day 30- MTA_5_** | **0** | **0** | **1** | **0** | **3** |
| **Day 30- MTA_6_** | **0** | **0** | **0** | **1** | **2** |
| **Day 30- MTA_7_** | **0** | **0** | **0** | **1** | **2** |
| **Day 30- MTA_8_** | **0** | **0** | **0** | **1** | **2** |
| **Day 30- MTA_9_** | **0** | **0** | **0** | **1** | **2** |
| **Day 30- MTA_10_** | **0** | **0** | **0** | **0** | **3** |

| **Scores of tissue reactions for MTA-ABS group on day-30** | **INFLAMMATION** | **NECROSIS** | **FOREIGN BODY REACTION (FBR)** | **FIBROSIS** | **NEW BONE FORMATION (NBF)** |
| --- | --- | --- | --- | --- | --- |
| **Day 30- MTA-ABS_1_** | **0** | **0** | **0** | **1** | **2** |
| **Day 30- MTA-ABS_2_** | **0** | **0** | **0** | **0** | **2** |
| **Day 30- MTA-ABS_3_** | **0** | **0** | **0** | **0** | **2** |
| **Day 30- MTA-ABS_4_** | **0** | **0** | **1** | **1** | **2** |
| **Day 30- MTA-ABS_5_** | **0** | **0** | **0** | **0** | **3** |
| **Day 30- MTA-ABS_6_** | **0** | **0** | **0** | **0** | **3** |
| **Day 30- MTA-ABS_7_** | **0** | **0** | **0** | **1** | **2** |
| **Day 30- MTA-ABS_8_** | **0** | **0** | **0** | **0** | **2** |
| **Day 30- MTA-ABS_9_** | **0** | **0** | **0** | **0** | **2** |
| **Day 30- MTA-ABS_10_** | **0** | **0** | **1** | **1** | **2** |

| **Scores of tissue reactions for CONTROL group on day-30** | **INFLAMMATION** | **NECROSIS** | **FOREIGN BODY REACTION (FBR)** | **FIBROSIS** | **NEW BONE FORMATION (NBF)** |
| --- | --- | --- | --- | --- | --- |
| **Day 30- EMPTY TUBE_1_** | **0** | **0** | **0** | **0** | **3** |
| **Day 30- EMPTY TUBE_2_** | **0** | **0** | **0** | **0** | **3** |
| **Day 30- EMPTY TUBE_3_** | **0** | **0** | **0** | **1** | **2** |
| **Day 30- EMPTY TUBE_4_** | **0** | **0** | **0** | **1** | **2** |
| **Day 30- EMPTY TUBE_5_** | **0** | **0** | **0** | **1** | **2** |
| **Day 30- EMPTY TUBE_6_** | **0** | **0** | **0** | **0** | **1** |
| **Day 30- EMPTY TUBE_7_** | **0** | **0** | **0** | **0** | **3** |
| **Day 30- EMPTY TUBE_8_** | **0** | **0** | **0** | **0** | **3** |

| **Scores of tissue reactions for MTA group on day-60** | **INFLAMMATION** | **NECROSIS** | **FOREIGN BODY REACTION (FBR)** | **FIBROSIS** | **NEW BONE FORMATION (NBF)** |
| --- | --- | --- | --- | --- | --- |
| **Day 60- MTA_1_** | **0** | **0** | **0** | **0** | **3** |
| **Day 60- MTA_2_** | **0** | **0** | **0** | **0** | **3** |
| **Day 60- MTA_3_** | **0** | **0** | **0** | **0** | **3** |
| **Day 60- MTA_4_** | **0** | **0** | **0** | **0** | **2** |
| **Day 60- MTA_5_** | **0** | **0** | **0** | **1** | **2** |
| **Day 60- MTA_6_** | **0** | **0** | **0** | **0** | **3** |
| **Day 60- MTA_7_** | **0** | **0** | **0** | **0** | **3** |
| **Day 60- MTA_8_** | **0** | **0** | **0** | **0** | **3** |
| **Day 60- MTA_9_** | **0** | **0** | **0** | **0** | **3** |
| **Day 60- MTA_10_** | **0** | **0** | **0** | **0** | **2** |

| **Scores of tissue reactions for MTA-ABS group on day-60** | **INFLAMMATION** | **NECROSIS** | **FOREIGN BODY REACTION (FBR)** | **FIBROSIS** | **NEW BONE FORMATION (NBF)** |
| --- | --- | --- | --- | --- | --- |
| **Day 60- MTA-ABS_1_** | **0** | **0** | **0** | **0** | **2** |
| **Day 60- MTA-ABS_2_** | **0** | **0** | **0** | **0** | **3** |
| **Day 60- MTA-ABS_3_** | **0** | **0** | **0** | **0** | **3** |
| **Day 60- MTA-ABS_4_** | **0** | **0** | **0** | **0** | **2** |
| **Day 60- MTA-ABS_5_** | **0** | **0** | **0** | **1** | **3** |
| **Day 60- MTA-ABS_6_** | **0** | **0** | **1** | **0** | **3** |
| **Day 60- MTA-ABS_7_** | **0** | **0** | **0** | **0** | **2** |
| **Day 60- MTA-ABS_8_** | **0** | **0** | **0** | **0** | **3** |
| **Day 60- MTA-ABS_9_** | **0** | **0** | **0** | **0** | **3** |
| **Day 60- MTA-ABS_10_** | **0** | **0** | **0** | **0** | **2** |

| **Scores of tissue reactions for CONTROL group on day-60** | **INFLAMMATION** | **NECROSIS** | **FOREIGN BODY REACTION (FBR)** | **FIBROSIS** | **NEW BONE FORMATION (NBF)** |
| --- | --- | --- | --- | --- | --- |
| **Day 60- EMPTY TUBE_1_** | **0** | **0** | **0** | **0** | **2** |
| **Day 60- EMPTY TUBE_2_** | **0** | **0** | **0** | **0** | **2** |
| **Day 60- EMPTY TUBE_3_** | **0** | **0** | **0** | **0** | **2** |
| **Day 60- EMPTY TUBE_4_** | **0** | **0** | **0** | **1** | **2** |
| **Day 60- EMPTY TUBE_5_** | **0** | **0** | **0** | **0** | **3** |
| **Day 60- EMPTY TUBE_6_** | **0** | **0** | **0** | **1** | **3** |
| **Day 60- EMPTY TUBE_7_** | **0** | **0** | **0** | **0** | **2** |
| **Day 60- EMPTY TUBE_8_** | **0** | **0** | **0** | **0** | **2** |

| **Scores of tissue reactions for MTA group on day-90** | **INFLAMMATION** | **NECROSIS** | **FOREIGN BODY REACTION (FBR)** | **FIBROSIS** | **NEW BONE FORMATION (NBF)** |
| --- | --- | --- | --- | --- | --- |
| **Day 90- MTA_1_** | **0** | **0** | **0** | **1** | **3** |
| **Day 90- MTA_2_** | **0** | **0** | **0** | **0** | **3** |
| **Day 90- MTA_3_** | **0** | **0** | **0** | **0** | **3** |
| **Day 90- MTA_4_** | **0** | **0** | **0** | **0** | **3** |
| **Day 90- MTA_5_** | **0** | **0** | **0** | **0** | **3** |
| **Day 90- MTA_6_** | **0** | **0** | **0** | **0** | **3** |
| **Day 90- MTA_7_** | **0** | **0** | **0** | **1** | **3** |
| **Day 90- MTA_8_** | **0** | **0** | **0** | **0** | **3** |
| **Day 90- MTA_9_** | **0** | **0** | **0** | **0** | **3** |
| **Day 90- MTA_10_** | **0** | **0** | **0** | **0** | **3** |

| **Scores of tissue reactions for MTA-ABS group on day-90** | **INFLAMMATION** | **NECROSIS** | **FOREIGN BODY REACTION (FBR)** | **FIBROSIS** | **NEW BONE FORMATION (NBF)** |
| --- | --- | --- | --- | --- | --- |
| **Day 90- MTA-ABS_1_** | **0** | **0** | **0** | **1** | **3** |
| **Day 90- MTA-ABS_2_** | **0** | **0** | **0** | **1** | **3** |
| **Day 90- MTA-ABS_3_** | **0** | **0** | **0** | **0** | **3** |
| **Day 90- MTA-ABS_4_** | **0** | **0** | **0** | **0** | **3** |
| **Day 90- MTA-ABS_5_** | **0** | **0** | **0** | **0** | **3** |
| **Day 90- MTA-ABS_6_** | **0** | **0** | **0** | **1** | **3** |
| **Day 90- MTA-ABS_7_** | **0** | **0** | **0** | **1** | **3** |
| **Day 90- MTA-ABS_8_** | **0** | **0** | **0** | **1** | **3** |
| **Day 90- MTA-ABS_9_** | **0** | **0** | **0** | **0** | **3** |
| **Day 90- MTA-ABS_10_** | **0** | **0** | **0** | **0** | **3** |

| **Scores of tissue reactions for CONTROL group on day-90** | **INFLAMMATION** | **NECROSIS** | **FOREIGN BODY REACTION (FBR)** | **FIBROSIS** | **NEW BONE FORMATION (NBF)** |
| --- | --- | --- | --- | --- | --- |
| **Day 90- EMPTY TUBE_1_** | **0** | **0** | **0** | **1** | **3** |
| **Day 90- EMPTY TUBE_2_** | **0** | **0** | **0** | **1** | **3** |
| **Day 90- EMPTY TUBE_3_** | **0** | **0** | **0** | **0** | **3** |
| **Day 90- EMPTY TUBE_4_** | **0** | **0** | **0** | **0** | **3** |
| **Day 90- EMPTY TUBE_5_** | **0** | **0** | **0** | **1** | **3** |
| **Day 90- EMPTY TUBE_6_** | **0** | **0** | **0** | **1** | **3** |
| **Day 90- EMPTY TUBE_7_** | **0** | **0** | **0** | **1** | **3** |
| **Day 90- EMPTY TUBE_8_** | **0** | **0** | **0** | **1** | **3** |
